# Sample size and sample composition for constructing growth reference centiles

**DOI:** 10.1177/0962280220958438

**Published:** 2020-10-12

**Authors:** TJ Cole

**Affiliations:** UCL Great Ormond Street Institute of Child Health, London, UK

**Keywords:** Growth reference, GAMLSS, LMS method, sample size, anthropometry

## Abstract

Growth reference centile charts are widely used in child health to assess weight, height and other age-varying measurements. The centiles are easy to construct from reference data, using the LMS method or GAMLSS (Generalised Additive Models for Location Scale and Shape). However, there is as yet no clear guidance on how to design such studies, and in particular how many reference data to collect, and this has led to study sizes varying widely. The paper aims to provide a theoretical framework for optimally designing growth reference studies based on cross-sectional data. Centiles for weight, height, body mass index and head circumference, in 6878 boys aged 0–21 years from the Fourth Dutch Growth Study, were fitted using GAMLSS. The effect on precision of varying the sample size and the distribution of measurement ages (sample composition) was explored by fitting a series of GAMLSS models to simulated data. Sample composition was defined as uniform on the age*^λ^* scale, where *λ* was chosen to give constant precision across the age range. Precision was measured on the z-score scale, and was the same for all four measurements, with a standard error of 0.041 z-score units for the median and 0.066 for the 2nd and 98th centiles. Compared to a naïve calculation, the process of smoothing the centiles increased the notional sample size two- to threefold by ‘borrowing strength’. The sample composition for estimating the median curve was optimal for *λ*=0.4, reflecting considerable over-sampling of infants compared to children. However, for the 2nd and 98th centiles, *λ*=0.75 was optimal, with less infant over-sampling. The conclusion is that both sample size and sample composition need to be optimised. The paper provides practical advice on design, and concludes that optimally designed studies need 7000–25,000 subjects per sex.

## 1 Introduction

Growth reference centile charts are widely used in child health and paediatric clinical practice to assess measurements that vary by age. Commonly, they apply to child anthropometry such as weight or height, and they are usually constructed to be representative of a particular national or regional population.

The construction of reference centiles involves drawing a sample of measurements from the population of subjects across the relevant age range. Over 30 years ago, I proposed the LMS method ^1–3^ as a way to construct centiles from such data, and the LMS method has since been subsumed within the family of GAMLSS models, Generalized Additive Models for Location Scale and Shape.^4^ GAMLSS is a set of models that assume an underlying frequency distribution for the data, and then estimate each of the distribution moments, i.e. the mean or median, standard deviation (SD) or coefficient of variation (CV), and optionally skewness and kurtosis, in the form of smooth curves plotted against age. This ensures that the centile curves, which are functions of the moments, are themselves smooth.

Despite the wide availability of such centile charts, and the relative accessibility of software to construct them,^4^ there has been far less debate about how best to design reference centile studies, i.e. to decide how many and which reference data to collect. Two distinct design questions arise: how many measurements need to be collected, i.e. the sample size, and at what ages should they be collected, i.e. the sample composition?

On sample size, Healy^5^ showed that assuming a Normal distribution, a group of 1000 8-year-old children estimates the 3rd height centile with a standard error (SE) of 0.30 cm, while the SE for the 50th centile is somewhat smaller at 0.18 cm. On this basis, the 1972 Cuban Growth Study was designed to recruit 1000 boys and 1000 girls per year of age from 3 to 9 years, and 56,000 children overall.^6^ The Fourth Dutch Growth Study of 1997^7^ chose its sample size of 14,500 children to detect a 1.8 cm height difference compared to the previous Third Growth Study.^8^ The World Health Organization growth standard required at least 200 children per sex per three-month age group in the cross-sectional study, plus 200 per sex for the longitudinal study.^9^ Guo et al.^10^ used simulation to estimate the SE for the 97th centile when updating the United States growth reference. Their findings were complicated in that the SE was larger (a) for weight and BMI compared to stature; (b) for puberty compared to outside puberty for weight and BMI and (c) for boys compared to girls for weight and BMI.^10^

Sample composition, i.e. the proportion of measurements to collect at different ages, was considered in the design of several of these studies,^6–9^ while Cole^11^ discussed other aspects of growth reference study design. It is generally recognised that more measurements are needed in infancy than later in childhood, for the simple reason that growth is faster in infancy.

Despite this effort to optimise the design, dedicated growth reference studies of boys and girls from birth to adult have varied widely in size, from 14,500 for the Fourth Dutch Growth Study up to 55,000 for the Cuban and First Dutch growth studies, with a median of 17,000.^12^ One important reason for this heterogeneity is the lack of a convincing and generally accepted sample size calculation, which if it existed would encourage researchers to design more similarly sized studies.

In contrast, a recent paper by Heude et al.^12^ constructed growth charts for French children using routinely collected big data, with a sample of 1.5 million measurements from nearly 240,000 children, with a mean of 6.1 heights and 7.1 weights per child. This is an interesting concept where the large numbers ensure high precision, but the risks of sampling bias and measurement error are also likely to be increased, and the multiple measurements per child complicate the design.

The heterogeneity in sample size among dedicated growth studies is due to two particular design problems. The first is that measurements such as weight have a non-normal frequency distribution, where standard formulae for the SE of centiles (apart from the median) do not exist. Cole^2^ produced an approximate formula with the LMS method, expressed as a function of the three moment curves, but it failed to address the other problem, which is that the centiles are smoothed across age groups, and this materially affects the SE.

As already stated, GAMLSS defines the reference centiles as functions of smooth moment curves plotted against age, and this renders the concept of an age group meaningless. The curve is supported by data not only at specific ages but also at neighbouring ages, a process which ‘borrows strength’ across the age range. The act of smoothing the data has the effect of considerably increasing the age-specific sample size and hence the precision, but in a way that is hard to quantify. Cole^2^ for example suggested that the smoothing increases the notional sample size by a factor of two to three, though with no evidence to support it.

From this it is clear that researchers wanting to construct growth reference centiles lack the framework necessary to optimise their study’s sample size and sample composition. The aim of this paper is to provide a comprehensive design framework for such studies, using data from the Fourth Dutch Growth Study as a case study. The remit is restricted to studies with cross-sectional data where individuals are measured just once, as studies with multiple measurement occasions involve extra complexities that need their own treatment.

## 2 Methods

### 2.1 Data

The Fourth Dutch Growth Study, carried out in 1996–1997, measured 7482 boys and 7018 girls aged from 11 days to 21 years.^7^ The boys’ data, including height, weight, body mass index (BMI) and head circumference, have since been posted online by Professor Stef van Buuren as the open source dataset *boys7482* in his *AGD* (analysis of growth data) package^13^ for the *R* statistical language.^14^ This is a large and high quality dataset, with the data already cleaned, and its open source status is useful for what follows. Other papers have used the same data as examples: van Buuren the height centiles when developing his worm plot,^15^ and Rigby and Stasinopoulos the BMI^16^ and head circumference data for GAMLSS.^17,18^

### 2.2 Statistical analysis

The process of optimising a study’s design involves identifying and manipulating the variability associated with the outcome of interest. Here the interest is in one or more constructed centile curves, and the formal outcome measure of variability is the SE of the centile curve, which can itself be visualised as a curve plotted against age. The description of the analysis proceeds as follows: (a) calculating a centile and its SE with Normally distributed data; (b) constructing centile curves using GAMLSS; (c) estimating SE curves for the centiles; (d) optimising the SE curves by manipulating sample composition and sample size, and (e) discussing strategies for defining the sample composition.

### 2.3 Centiles based on the Normal distribution

For a single age group, and assuming an underlying Normal distribution, the mean and SE of the 100α'th centile are given by

(1) $$\begin{array}{l} m\mathrm{ean}=\mu +z_{\alpha }\sigma \\ SE=\sigma \sqrt{\left(1+z_{\alpha }{\;}^{2}/2\right)/n} \end{array}$$

respectively,^5,11^ where $z_{\alpha }$ is the normal equivalent deviate corresponding to centile 100α; *μ* and *σ* are the age-specific mean and SD, and *n* is the sample size. For the median (or 50th centile), $z_{\alpha }=0$, while for the 3rd and 97th centiles, $z_{\alpha }=\pm 1.88$. Thus, the SE for these outer centiles is $\sqrt{\left(1+1.88^{2}/2\right)}=1.66$ times or 66% larger than for the median, irrespective of *n*, and this increases to 93% larger for the 1st and 99th centiles (where $z_{\alpha }=\pm 2.33$), showing how the imprecision increases with the centile’s distance from the median.

Growth data are routinely age-standardised by converting them to z-scores:

(2) z=(y−μ)/σ

where *y* is the measurement and *z* is the z-score. Thus, mean centile $C_{100\alpha }$ (equation (1)) expressed as a z-score is $z_{\alpha }$ at all ages. Differentiating equation (2) gives δz=δy/σ, so to express the SE from equation (1) in z-score units it needs dividing by the SD; call it $SE_{z}$ where

(3) $$SE_{z}=\sqrt{\left(1+z_{\alpha }{\;}^{2}/2\right)/n}$$

or in its logged form

(4) $$\log SE_{z}=\left[\log\left(1+z_{\alpha }{\;}^{2}/2\right)-\log(n)\right]/2$$

$SE_{z}$ is the outcome measure on which the paper focuses.

For the median, equation (3) simplifies to $SE_{z}=\sqrt{1/n}$, so for example Healy’s group of 1000 8-year-olds estimates median height with $SE_{z}=\sqrt{1/1000}=0.032$ z-scores, and for $z_{\alpha }=\pm 2$ (corresponding to rounded 2nd and 98th centiles, see later) with $SE_{z}=\sqrt{(1+2^{2}/2)/1000}=\sqrt{3/1000}=0.055$. For design purposes, equation (3) can be rearranged as follows

(5) $$n=\left(1+z_{\alpha }{\;}^{2}/2\right)/SE_{z}{\;}^{2}$$

One can then specify the size of $SE_{z}$ required for the chosen centile, and equation (5) gives the required sample size *n*. Note that equations (3), (4) and (5) are independent of *μ* and *σ* so they apply to any measurement, be it height or weight or whatever. This is a useful simplification.

### 2.4 Centile curve construction

The GAMLSS centile models developed by Stasinopoulos and Rigby^4^ are extensions of the Normal distribution. For the LMS method, which is the most widely used of them,^3^ the extension is in the form of an adjustment for skewness. It estimates the first three moments of the measurement distribution as the age-varying median (*μ*), the CV (*σ*), and skewness in the form of a Box-Cox transformation (*λ*).^19^ The name LMS comes from the initials of *λ*, *μ* and *σ*.

Note that in the LMS method, *σ* refers to the generalised CV not the SD, which means that the corresponding generalised SD is the median times the CV, i.e. SD=μσ. In what follows, *σ* refers to the CV.

In GAMLSS, the LMS method is renamed the BCCG distribution, for Box-Cox Cole and Green, and the skewness parameter is called *ν* rather than *λ*.^4^ The underlying GAMLSS assumption is that after adjustment the measurement follows some pre-specified standard frequency distribution, which for the LMS method is the Normal distribution. Thus, the LMS method converts skew data to normally distributed z-scores. There are also two GAMLSS models that extend BCCG by adjusting for kurtosis *τ*, based respectively on the power exponential distribution (Box-Cox power exponential or BCPE)^16^ and the *t* distribution (Box-Cox *t* or BCT),^17^ where the distributions both have a *τ* parameter controlling the kurtosis. Thus, BCCG has three moment functions, while BCT and BCPE have four.

The algebra underlying all three models is the same, as follows

(6) $$C_{100\alpha }=\{\begin{array}{ll} \mu {\left(1+\nu \sigma z_{\alpha }\right)}^{1/\nu }, & \text{if }\nu \neq 0\\ \mu e^{\sigma z_{\alpha }}, & \text{if }\nu =0 \end{array}$$

where $C_{100\alpha }$ is the measurement centile corresponding to the underlying distribution’s equivalent deviate $z_{\alpha }$, while *μ*, *σ* and *ν* are respectively the median, CV and Box-Cox power. The reverse operation converts measurement *y* to z-score *z*, analogously to equation (2) for the Normal distribution

(7) $$z=\{\begin{array}{ll} \frac{{\left(y/\mu \right)}^{\nu }-1}{\nu \sigma }, & \text{if }\nu \neq 0\\ \frac{\log(y/\mu )}{\sigma }, & \text{if }\nu =0 \end{array}$$

Fitting the model, be it BCCG, BCT or BCPE, involves estimating the curves for *μ*, *σ*, *ν* and optionally *τ* as smooth functions of age. GAMLSS is implemented in *R* as the *gamlss* package, and it offers several functions for fitting smooth curves to data. The most useful are based on the penalised cubic B-splines or P-splines developed by Eilers and Marx.^20^ By default they have a basis of 20 equally spaced and automatically penalised knots, which simplify the spline curve chores of choosing the knot positions or degrees of freedom. The standard P-spline function in *gamlss* is *pb*(), while the variant *pbm*() constrains the curve to be monotonic (which is useful for the *μ* curve when it is known to increase monotonically), and *pbz*() is valuable for simple curves like *ν* or *τ*, selecting a constant value if it fits better than a linear trend.^21,22^ The description by Rigby and Stasinopoulos of their algorithm for estimating the P-spline degrees of freedom uses head circumference from the Fourth Dutch Growth Study as an example.^18^

For a BCCG model fitted to the *boys7482* weight data, the required *gamlss* call is as follows

(8) $$\begin{array}{l} \mathrm{wt\_BCCG\leq gamlss(wt∼pb}\left(\mathrm{age}\right)\mathrm{,sigma}\mathrm{.formula=pb}(\mathrm{age}),\mathrm{nu}\mathrm{.formula=pbz}\left(\mathrm{age}\right)\mathrm{,family=BCCG},\\ \mathrm{data=na}\mathrm{.omit(boys7482[,c('age','wt')])} \end{array}$$

And for a BCT model, the call is

(9) $$\begin{array}{l} \mathrm{wt\_BCT\leq gamlss(wt∼pb}\left(\mathrm{age}\right)\mathrm{,sigma}\mathrm{.formula=pb}(\mathrm{age}),\mathrm{nu}\mathrm{.formula=pbz}(\mathrm{age}),\\ \mathrm{tau}\mathrm{.formula=pbz}\left(\mathrm{age}\right)\mathrm{,family=BCT},\mathrm{data=na}\mathrm{.omit(boys7482[,c('age','wt')])} \end{array}$$

where wt is weight in kg and age is age in years. This is a powerful and flexible model, and good enough for many purposes, but it can be improved. The *μ* curve is usually steeper in infancy than in childhood, since growth velocity (for all the measurements discussed here) is high at birth and falls steeply during infancy. The fitted curve has to model this global curvature as well as more short-term trends, so there is benefit in minimising the curvature by transforming age, as suggested originally by Rao.^23^ This can be done by fitting the *μ* curve using pb(f(age)) where $f(\mathrm{age})=age^{\lambda }$ and *λ* < 1, e.g. √age or log age, and optimising *λ* by binary search in the region 0 ≥ *λ* ≥ 1 keeping the degrees of freedom constant. Rigby and Stasinopoulos^16^,^17^ call *λ* a hyper parameter (NB it is not the *λ* of the LMS method), and more recently they have named it *ξ* rather than *λ*. There is usually less benefit in fitting the *σ*, *ν* and *τ* curves to transformed age, and here they are fitted to age.

Alternative GAMLSS models can be compared using the Bayesian Information Criterion (BIC), which penalises complexity such that the curve with minimal BIC tends to be optimal, i.e. neither under- nor over-smoothed. Rigby and Stasinopoulos^16^ prefer to use the generalised Akaike Information Criterion GAIC(3), which for large datasets like *boys7482* imposes a smaller penalty than the BIC. The worm plot diagnostic developed by van Buuren and Fredriks^15^ is also helpful to test the fit of the model across the age range.

As already stated, BCT and BCPE model kurtosis in addition to skewness. It might seem good practice to model kurtosis, but it is present only in the extreme tails of the distribution, if at all, and as such it affects the distribution only beyond say the 1st and 99th centiles. If the range of centiles on the growth chart is going to be less extreme than this, e.g. from the 3rd to the 97th centile, then there is little point in modelling kurtosis *even if it is present*, as it unnecessarily over-complicates the model.

Thus, for many purposes, the simpler BCCG model provides an adequate fit. To make the judgement, one should superimpose plots of the required centiles for the models with and without kurtosis adjustment, and see to what extent the centiles differ – any differences will tend to be in the outer centiles. For this reason, the simulations described in later sections are carried out using BCCG rather than BCT or BCPE.

### 2.5 Estimation of the centile standard error curve

The fitted GAMLSS model provides estimates of the moment curves with their SE curves, and the SE curves are exact being based on the underlying P-splines. The *μ* curve is the estimate of the 50th centile, so if the precision of the 50th centile is to be the criterion used to design the study, then the SE band for the *μ* curve is the appropriate summary statistic to use, adjusted for age as $SE_{z}$ (equation (3)).

For centiles other than the 50th, the SE bands need to be obtained via the bootstrap, since the centile curves are nonlinear functions of the moment curves (equation (6)). The bootstrap process involves repeatedly drawing samples of the data with replacement, refitting the model and saving the moment curves. These sets of curves each provide a separate estimate of any required centile, and the SD across these centile estimates at each age provides the centile's bootstrapped SE curve. To derive the SE as $SE_{z}$, the variability across estimates needs to be calculated on the z-scores of the centiles (equation (7)) based on the original GAMLSS model’s moment curves.

The SE is in general larger for centiles above than below the median, simply because the centile itself is larger above the median. However, in z-score terms, $SE_{z}$ for pairs of centiles symmetric about the median should be very similar, because formulae (3) and (5) are functions of $z_{\alpha }{\;}^{2}$ and hence are independent of $\mathrm{sign}(z_{\alpha })$.

### 2.6 Optimal study design based on the standard error curve

As already stated, the appropriate summary statistic to optimise the study design is the z-score standard error curve $SE_{z}$ for some pre-specified centile (e.g. the 50th or 2nd or 99th). Ages where $SE_{z}$ is relatively large indicate that extra data are needed there. By notionally adding extra data, one can reduce the error in that region, and by iteration effectively constrain $SE_{z}$ to a constant value across the age range. This suggests an important design principle – the optimal $SE_{z}$ curve should be *flat*. The Cuban Growth Study stated this same principle: ‘Population standards should be estimated with the same accuracy at each age’.^6^

The question is, how to do this? The answer is to use simulation to explore a series of different study designs, all with the same sample size, and iterate to find the optimum. The key requirement is a pre-existing or base GAMLSS model on which to build the simulations, and the open source nature of the *boys7482* dataset makes it ideal for the purpose. The steps are as follows:

- specify the ages at which measurements are to be made, as described in the next section;
- simulate measurements at these ages, by generating uniformly distributed random proportions α (corresponding to centiles 100α) for each age and converting them to z-scores and then measurements by applying equation (6) to the base model;
- update the base model using the simulated data;
- inspect the selected $SE_{z}$ curve;
- repeat (a) to (d) as necessary until the $SE_{z}$ curve is essentially flat.

### 2.7 Specifying the sample composition

The first step of the process is to decide on the ages of measurement to be used, in other words to define the sample composition as summarised by the shape of the age distribution. Note that this is independent of the sample size, as the numbers at each age can be scaled up or down as required. The Cuban Growth Study has useful advice on sample composition: ‘At earlier ages and in adolescence, the fact that growth is faster implies that a larger sample (effectively a more frequent age sampling) is needed’.^6^ This makes two distinct points: (a) that the number of sample points at a particular age should be proportional to the growth velocity at that age, so that the histogram of age should be the same shape as the growth velocity curve; and (b) that there are two distinct ways to define the sample, depending on whether the study design is cross-sectional or longitudinal.

Cross-sectional studies consist of a series of age groups. The Cuban Growth Study for example had 27 groups, starting with 0–4, 4–8 and 8–12 months and ending with 16.5–17.5, 17.5–18.5 and 18.5–20.0 years, each with their own target sample size. In longitudinal (cohort) studies by contrast, children are measured repeatedly at a series of design ages. For example, the World Health Organization Multicentre Growth Reference Study ‘enrolled [infants] at birth and measured [them] at home 21 times, at weeks 1, 2, 4 and 6; monthly from 2 to 12 months; and every two months in the second year’.^9^ Design ages being closer together are equivalent to oversampling. These two designs can be made more similar by treating the mid-age in each group as the design age, then they differ only in the age range within each group, which is by definition non-zero in a cross-sectional study and zero (at least nominally) in a cohort study.

Once the ages/age groups have been set, the numbers of measurements for each group need to be specified, so as to define the overall sample size. For longitudinal studies, the groups will all be the same size (i.e. that of the cohort, though possibly including an adjustment for dropout), whereas for cross-sectional studies they may differ. Also in cross-sectional studies, the ages within each group need to be simulated as random uniform deviates within the group’s age range, whereas in longitudinal studies the ages are the design ages.

It is worth explaining here why longitudinal studies are more complicated to design than cross-sectional studies, and hence outside the paper’s remit. Their repeated measures can impact on both the precision and accuracy of the centiles,^24,25^ and this adds complexity to the optimisation process. For example, if individuals tend to be measured more often when they are sick, this can bias the lower centiles.^24^ However, longitudinal studies as described here involve (most) subjects being measured at (nearly) all design ages, so the design is close to balance and the centiles are unlikely to be very biased. But against that, even with a balanced design, the repeated measures being correlated reduce the information content of the data, increase the imprecision and inflate the $SE_{z}$ curve. In addition, the exact nature of this loss of precision depends on the correlation structure, which is a complex function of the mean and difference of each correlation’s two ages of measurement.^26,27^ It is for these reasons that longitudinal studies are excluded from further consideration here.

An alternative and quite different way to specify the sample composition is to not group age at all, but to simulate the measurement ages across the whole range. This involves: (a) defining a suitable monotonic sampling function f(age); (b) sampling from a uniform distribution in the range f(min⁡(age)) to f(max⁡(age)) and (c) back-transforming using the inverse function $f^{-1}(\mathrm{age})$ to obtain the required ages. An example function might in theory be based on the growth velocity curve as mentioned above, though its lack of monotonicity rules it out.

Instead it turns out – and this is one of the key insights of the paper – that the already familiar function $f(\mathrm{age})=age^{\lambda }$ is useful here, where $f^{-1}(\mathrm{age})=age^{1/\lambda }$. It corresponds to sampling uniformly on the $age^{\lambda }$ scale, and in the simplest case λ=1, so $f(\mathrm{age})=f^{-1}(\mathrm{age})=\mathrm{age}$, leading to equal numbers across the range (ignoring sampling error). But for λ<1, it leads to a distribution that over-samples at younger ages – a common requirement – and the smaller *λ* is, the greater the over-sampling.

$SE_{z}$ is inevitably larger at the extremes of the age range because there are no data outside the range. So the $SE_{z}$ curve is higher at the ends than in the middle. This can be managed either by over-sampling the youngest and oldest age groups, or by sampling beyond the limit and truncating the resulting curves^11^ (though the latter approach obviously does not work for birth data). The WHO growth standard for example collected data from birth to six years and published the charts to five years.^28^

To help with visualisation, the $SE_{z}$ curves are summarised as linear trends on age, based on data restricted to 2–18 years (at 0.1 year intervals) to minimise edge effects. The effects of centile, age, *λ* and measurement on $\log SE_{z}$ are explored using analysis of variance and multiple regression.

### 2.8 Software

The analysis is done using *R* version 3.6.3,^14^ with the modelling in *gamlss* version 5.1–6^4^ and the plots created in *ggplot2* version 3.3.0.^29^ The appendix includes *R* code for the functions *optimal_design* and *nagegp*.

## 3 Results

The *boys7482* dataset has complete data on weight, height, BMI and head circumference for 6878 boys, and this restricted dataset is used for all the base GAMLSS models. They are fitted as equations (8) and (9) for BCCG and BCT, respectively, except with age*^λ^* for the *μ* curve. Table 1 summarises the BCCG and BCT models fitted to the four measurements, which are compared using the BIC, where the BIC is expressed as the difference relative to the BIC for the optimal model. BCT fits consistently better than BCCG, most obviously with head circumference and BMI, while BCPE lies in between (not shown). The equivalent degrees of freedom (edf) for the spline curves are relatively large for *μ*, indicating the complex shape of the median curves, whereas the edf for the *σ*, *ν* and *τ* curves are progressively smaller. The integers 1 and 2 in the table indicate respectively a constant value and a straight line for the fitted curve. The optimal age powers *λ* are 0.75 for weight, 0.5 (i.e. square root) for height and 0.31 (close to cube root) for both BMI and head circumference. The otherwise equivalent models with λ=1 for *μ* fit appreciably less well. Conversely, fitting the *σ* curve on the age^λ^ scale makes little difference, changing BIC by respectively −5, +5, −6 and 0 units for weight, height, BMI and head circumference.

**Table 1.** Summary statistics for BCCG and BCT models fitted to boys’ weight, height, BMI and head circumference: Fourth Dutch Growth Study (*n *=* *6878).

	Weight		Height		BMI		Head circumference	
	BCCG	BCT	BCCG	BCT	BCCG	BCT	BCCG	BCT
*μ* edf	14.2	14.2	16.0	16.1	12.2	12.3	12.7	13.0
*σ* edf	8.3	9.3	7.8	8.0	5.5	6.7	2.0	3.3
*ν* edf	4.5	5.2	2^a^	3.3	3.6	6.1	2^a^	2^a^
*τ* edf	–	1^b^	–	1.8	–	2^a^	–	2^a^
*λ*	0.75		0.50		0.31		0.31	
BIC	32	0	1	0	47	0	214	0
BIC for *λ* = 1	76	45	67	51	137	86	300	95

a Curve is a straight line.

b Curve is a constant.

edf: equivalent degrees of freedom; BIC: Bayesian information criterion (relative to the minimum, which is set to zero).

**Figure 1.**
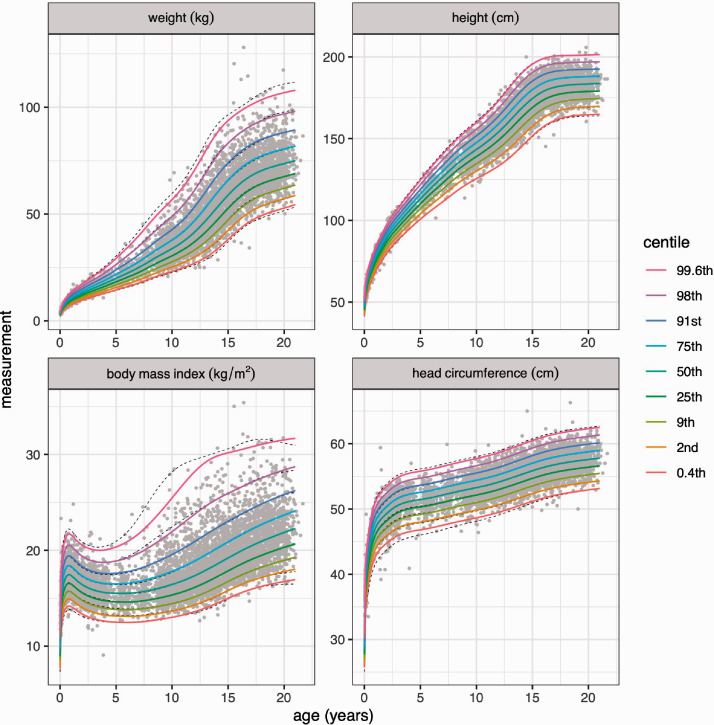
Growth reference centiles for boys weight, height, body mass index and head circumference from the Fourth Dutch Growth Survey (*n *=* *6878). The nine centiles, spaced two-thirds of a z-score apart, are estimated by GAMLSS with the BCCG model (coloured lines) and the BCT model (dashed lines). The raw data are shown in grey.

**Figure 2.**
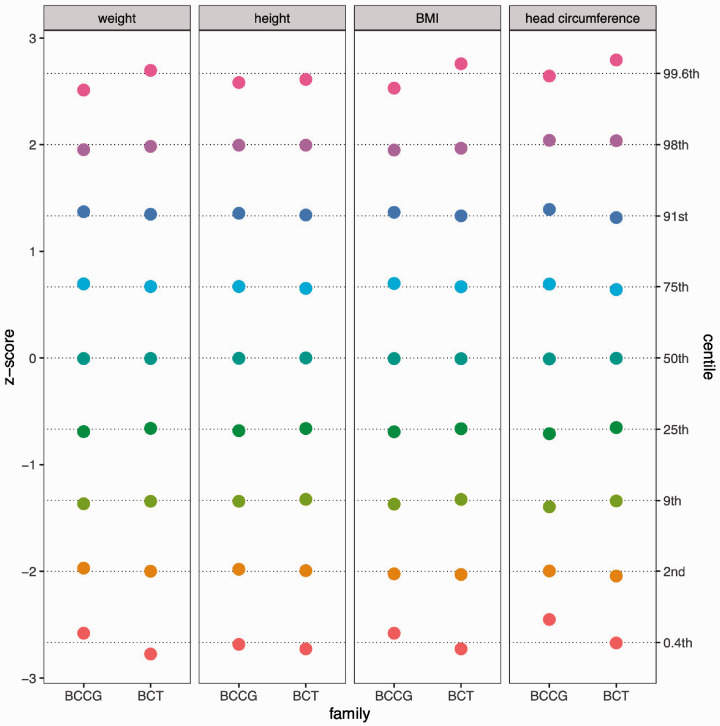
Goodness of fit for the eight models and nine centiles in Figure 1, where each point indicates the z-score and centile corresponding to the nominal centile across all ages.

Figure 1 shows the data and nine centile curves for the eight models, with the BCCG centiles in colour and the BCT centiles in grey (dashed lines). The nine centiles are spaced two-thirds of a z-score apart and extend from the 0.4th to the 99.6th centile.^30^ In particular, the centiles corresponding to z = ±2 (the 2.3rd and 97.7th centiles) are for simplicity called the 2nd and 98th centiles. The main differences between the BCCG and BCT models lie in the 0.4th and 99.6th centiles (z = ±2.67), which are further apart with BCT, particularly for BMI and head circumference, and they indicate the presence of leptokurtosis or heavy tails; conversely, the seven inner centiles are on the whole very similar. Figure 2 confirms this by plotting the observed z-scores and centiles for each model corresponding to the expected centiles across all ages. BCCG and BCT both provide a good fit to the inner centiles, while for the outer centiles, BCT usually – though not always – fits better than BCCG.

**Figure 3.**
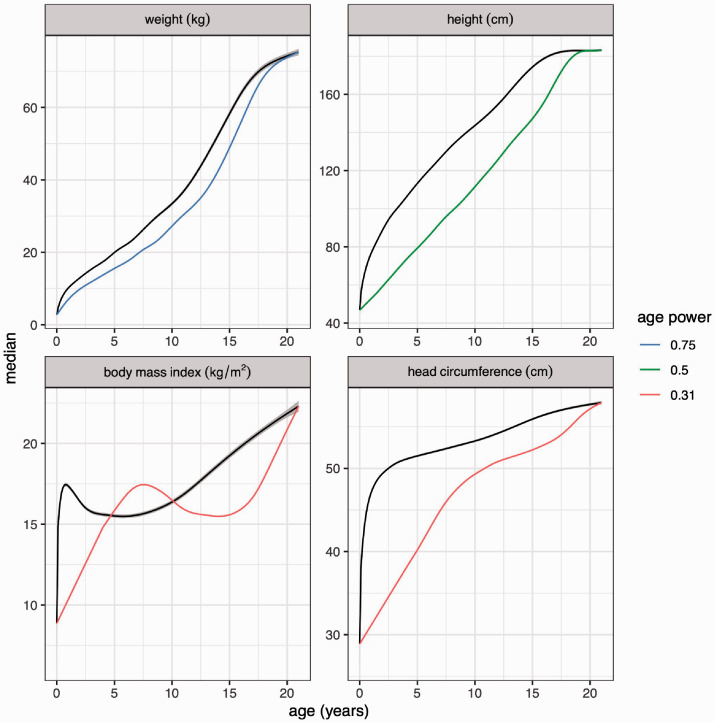
Median curves with 95% confidence bands for boys weight, height, body mass index and head circumference from Figure 1 (black), and the same curves plotted against *age^λ^ x max*(*age*)^1–λ^ with the optimal *λ* for each measurement from Table 1 (colour).

Initially the focus is on the median curve. Figure 3 shows the median curves for the four measurements (in black), along with their 95% confidence bands. On the whole, the bands are narrow, they increase with increasing age, and they are narrower for height and head circumference than for weight and particularly BMI. Figure 3 also shows in colour how the shapes of the median curves change when plotted against age transformed to $age^{\lambda }\times age_{\max}{\;}^{1-\lambda }$, i.e. as modelled taking f(age) into account (the $age_{\max}{\;}^{1-\lambda }$ multiplier rescales age). The value of *λ* for each measurement reflects the steepness of the (black) median curve in infancy, being much steeper for BMI and head circumference than for weight or height. Note too that the blue weight curve and particularly the green height curve are closer to linear throughout childhood on the transformed age scale, and the impact of the pubertal growth spurt is correspondingly reduced.

### 3.1 Z-score standard error curves for the median curve

**Figure 4.**
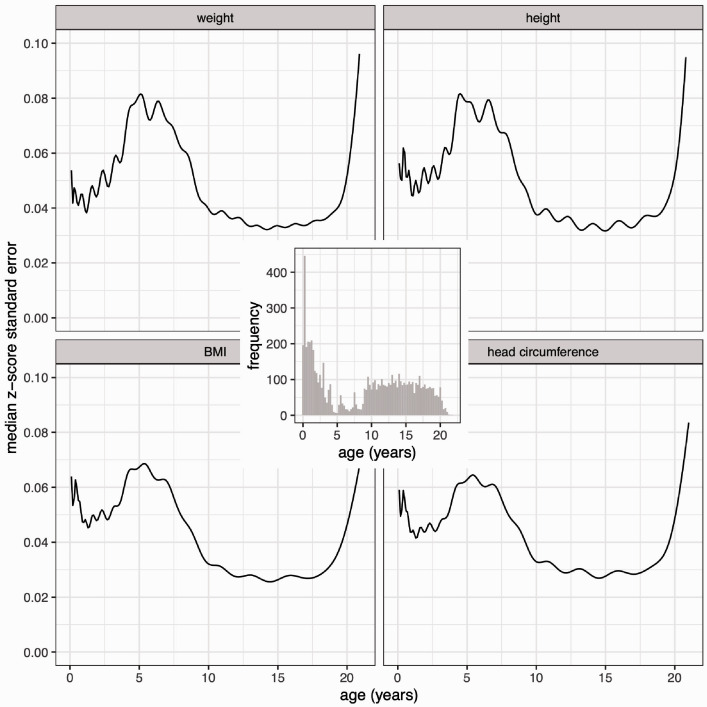
Standard error curves for the four measurement median curves of Figures 1 and 2, calculated on the z-score scale, and (inset) the age distribution of the underlying data.

The confidence bands for the median curves in Figure 3 vary subtly by age, and to see them better, Figure 4 shows the standard error curves as measured on the z-score scale. The transformation makes all four $SE_{z}$ curves strikingly similar in shape, with values between 0.03 and 0.08 z-score units except at the extremes of age. This accords with equation (3) that $SE_{z}$ should be broadly similar for the different measurements. Nevertheless, it is a surprise to see how uniform the curves are.

Ignoring the edge effects, $SE_{z}$ is low in infancy and in later childhood but higher in mid-childhood. Also shown in Figure 4 (inset) is the age distribution of the measurements, with infancy over-sampled compared to later in childhood, and relatively few data from three to nine years. The peak in $SE_{z}$ clearly corresponds to the under-sampled region, confirming the principle that data density and $SE_{z}$ are inversely related (equation (3)). In addition, the over-sampling in infancy is seen to be necessary, as even with it present $SE_{z}$ is larger in infancy than in later childhood.

**Figure 5.**
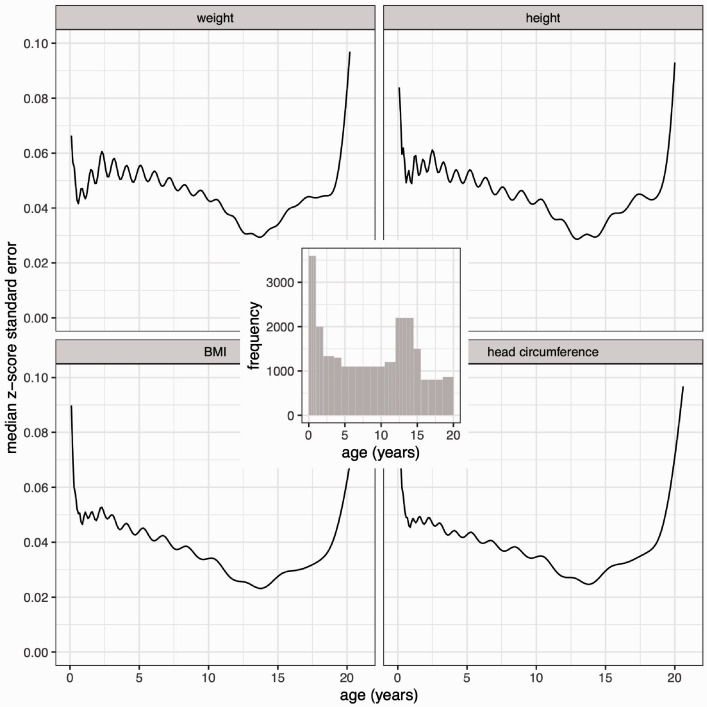
Z-score standard error curves for the four measurement median curves based on simulated data sampled from the Cuban Growth Study’s planned age distribution (inset).

If the sample composition is to be optimised, how should the age distribution in Figure 4 be modified? Clearly the three to nine year gap needs filling, but does puberty with its higher growth velocity also need to be over-sampled? The Cuban Growth Study^6^ assumed that it did, and was designed around the height velocity curve with peaks of over-sampling both in infancy and puberty as shown in Figure 5 (inset); it shows the planned numbers of boys in 27 age groups. A simulated dataset the same size as *boys7482* was sampled from this planned age distribution, and the body of Figure 5 shows the resulting $SE_{z}$ curves for the four measurement median curves, which are directly comparable to those in Figure 4.

All four curves are again similar in shape. They are also flatter than in Figure 4, but they fall with age from infancy to a minimum at age 14 that corresponds to the peak of pubertal over-sampling. The extra numbers in puberty have lowered the curve, yet what is required is a flat curve, so the pubertal over-sampling has over-compensated and hence is inefficient.

**Figure 6.**
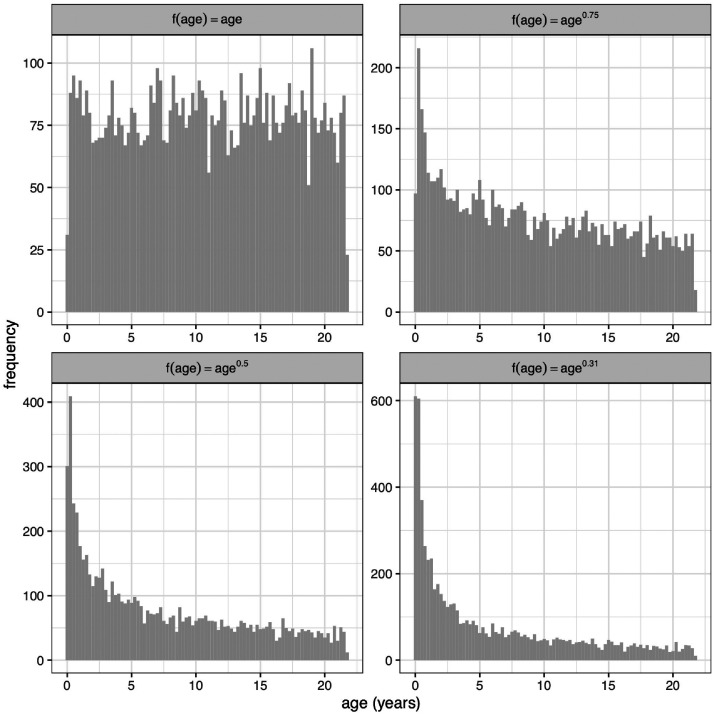
Random samples of age (*n *=* *6878) for sampling schemes based on age raised to the power 1, 0.75, 0.5 and 0.31 (from Table 1). See text for details.

If the pubertal peak is omitted from the design in Figure 5, the distribution becomes a monotonically falling pattern from infancy through childhood. This it turns out is a pattern that can be generated by sampling uniformly on $f(\mathrm{age})=age^{\lambda }$ with *λ* < 1. Figure 6 shows examples of the resulting distributions for uniform age (*λ* = 1) and the optimal *λ* values of 0.75, 0.50 and 0.31 from Table 1, where infancy is progressively more over-sampled as *λ* falls in value.

**Figure 7.**
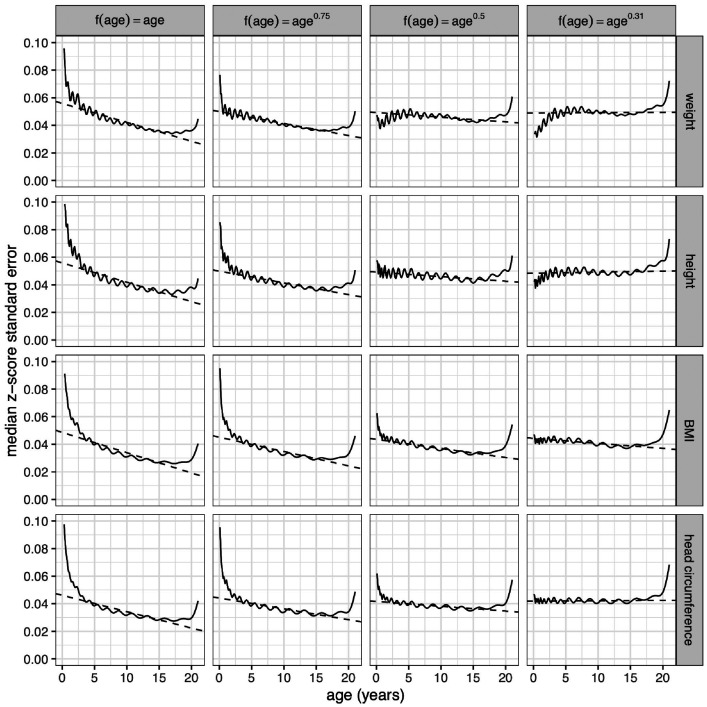
Z-score standard error curves for median boys weight, height, body mass index and head circumference using simulated data with sampling schemes based on age raised to the power *λ* = 1, 0.75, 0.5 and 0.31. The linear trends of the curves (dashed lines) summarise the data from 2 to 18 years.

Figure 7 repeats Figures 4 and 5 in showing $SE_{z}$ curves for the four measurement median curves, with data simulated by sampling using $f(\mathrm{age})=age^{\lambda }$ and the four *λ* values in Figure 6. Looking first at the uniform age distributions in the left column, all four $SE_{z}$ curves are high in infancy and fall steeply through childhood until age 18. This shows the imprecision that arises in infancy if it is not over-sampled. Looking to the right along each row, *λ* falls and the curves become progressively lower in infancy due to the over-sampling there, and closer to flat overall. The optimal *λ* values for all four measurements are those where the curves are effectively flat, i.e. between 0.31 and 0.5. This represents a considerable degree of infant over-sampling, as Figure 6 confirms, and it supports current practice for growth studies to collect measurements more frequently in infancy than later in childhood.

### 3.2 Z-score standard error curves for bootstrapped centile curves

**Figure 8.**
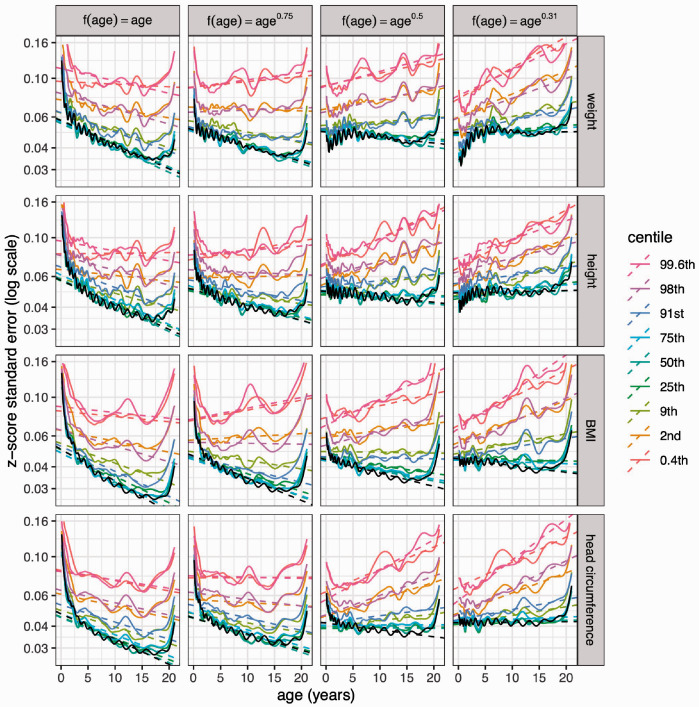
Z-score standard error curves bootstrapped for the nine centiles of Figure 1, by measurement and age power *λ* as in Figure 6, plotted on a log scale. The curves for the median from Figure 7 are also shown in black. The linear trends of the curves (dashed lines) summarise the data from 2 to 18 years.

The results thus far relate to the median curve, which is not necessarily the best centile to use for the sample size calculation. Figure 8 extends Figure 7 by showing bootstrapped $SE_{z}$ curves (now on a log scale) for the nine centiles of Figures 1 and 2 (in colour) plus the median $SE_{z}$ curves (in black) from Figure 7, along with summaries of each curve as a dashed line. Each bootstrapped curve is based on 500 bootstrap samples, and the data for each measurement are simulated with the four sample composition patterns of Figure 6. There are six striking features of Figure 8:

1. Apart from edge effects, the curves are all broadly linear.
2. The coloured bootstrapped curves are noisier than the black median curves.
3. The coloured and black median curves are otherwise very similar in shape.
4. The pairs of centile curves that are equally spaced about the median, i.e. the 25th/75th, 9th/91st, 2nd/98th and 0.4th/99.6th centiles, are very close to each other.
5. The further the centiles are from the median, the greater their curve intercept.
6. Within each facet, the outer centile curves are progressively steeper in slope than the median curve – this is entirely unexpected.

The first point indicates that basing the sample composition on age*^λ^* works well, in that it provides a linear $SE_{z}$ curve which can be made flat by suitable choice of *λ*. The second point is unsurprising given the two types of estimation. Points 3 to 5 show that $SE_{z}{\;}^{2}$ increases monotonically with $z_{\alpha }{\;}^{2}$ as in equation (4). The final point 6 also relates $SE_{z}$ to $z_{\alpha }{\;}^{2}$, but in terms of the slope of the curve on age.

To explore further the association between $SE_{z}$ and $z_{\alpha }{\;}^{2}$ by age, the data for $\log SE_{z}$ underlying the curves in Figure 8 are summarised by analysis of variance. Each facet contains nine bootstrapped centile curves, here referred to as *z*, and there are facets for the four measurements *y* (i.e. weight, height, etc.) by four *λ* values; the full model is $\log SE_{z}∼\mathrm{age}*z*\lambda *y$. Each curve is restricted to age 2–18 at 0.1 year intervals to avoid edge effects, i.e. 161 points per curve or 161×9×4×4= 23,184 points altogether. Table 2 shows two versions of the analysis of variance table, pared down to highlight the largest components of variance. On the left, *z* and *λ* are represented as respectively nine-level and four-level factors, while on the right they are continuous vectors, with *z* fitted as the function $\log\left(1+z^{2}/2\right)$ from equation (4). The two models fit well, explaining respectively 97% and 92% of the variance. By far the largest term is *z*, accounting for 77% of the total as a factor and 74% – only slightly less – as a continuous variable. This confirms that $\log SE_{z}$ is highly correlated with $\log\left(1+z^{2}/2\right)$ as equation (4) predicts.

**Table 2.** Analysis of variance tables for the model $\log SE_{z}∼\mathrm{age}*z*\lambda *y$ fitted to the bootstrapped *SE_z_* curves in Figure 8, where *z* represents the centiles, either as a nine-level factor or the vector $\log(1+z^{2}/2)$ from equation (4); *y* is a four-level factor for the measurements, *λ* is the age power (either as a four-level factor or vector) and *age* is in years.

	z and *λ* as factors		z and *λ* as vectors	
Model term	d.f.	Sum of squares	d.f.	Sum of squares
Age	1	0	1	0
z	8	2162	1	2077
*λ*	3	159	1	148
y	3	148	3	148
Age : z	8	72	1	71
Age : *λ*	3	123	1	122
z : *λ*	24	1	1	0
Age : y	3	1	3	1
z : y	24	27	3	15
*λ* : y	9	6	3	3
Age : z : *λ*	24	3	1	2
Age : z : y	24	4	3	1
Age : *λ* : y	9	3	3	0
z : *λ* : y	72	5	3	0
Age : z : *λ* : y	72	3	3	1
Residual	22,896	90	23,152	216
Total	23,183	2806	23,183	2806
R^2^		0.968		0.923
Residual SD		0.063		0.097

Note: Age is restricted to 2–18 years to minimise edge effects. Two separate models are shown, with factors (left) and vectors (right). The terms explaining most variance are *z*, *λ*, *y*, age:z and age:λ. The vector forms of *z* and *λ* fit almost as well as the factors.

The flat curve principle requires $\log SE_{z}$ to be independent of age, and by chance the *age* main effect is very close to zero. In addition, the age:y interaction is small and can be ignored, but age:z and age:λ are relatively large. This means that to constrain the age slope to zero, *z* and *λ* need to take values that ensure their interactions cancel out the main age effect.

Table 2 shows that age:z and age:λ fit equally well as factors or vectors, so the continuous model (right) is used. In addition, *y* is dropped from the model because the optimal design should generalise to all measurements. Furthermore, BMI is excluded from the data as it is a function of weight and height, and as such should not be double-counted.

The simplified model is $\log SE_{z}∼\mathrm{age}*(\log(1+z^{2}/2)+\lambda )$, and the regression results are shown in Table 3. For the design to be optimal, the three age terms need to sum to zero. This is achieved for a pre-specified *z* value by calculating *λ* appropriately. For example, if z=0 (i.e. the median curve) then the age:z term vanishes, and to give a zero age slope, *λ* must be minus the *age* term divided by the age:λ term, i.e. λ=−0.231/−0.0604=0.38. This chimes with Figures 7 and 8 where the median curves are close to flat for λ=0.31. In general, optimal $\lambda =(0.0231+0.0205(\log(1+z^{2}/2))/0.0604$. Once *z* and *λ* are set, the first three terms of Table 3 give $SE_{z}$ for that design. For example, with z=0 and λ=0.38, the *z* term vanishes and $SE_{z}=\exp(-3.088-0.283\times 0.38)=0.041$. This is similar to $SE_{z}$ for the median curves with λ=0.31 in Figures 7 and 8.

**Table 3.** Regression results for the model $\log SE_{z}∼\mathrm{age}*(\log(1+z^{2}/2)+\lambda )$ fitted to the bootstrapped *SE_z_* curves for weight, height and head circumference in Figure 8.

Model term	Regression coefficient	Standard error
Constant	−3.088	0.0029
$\log\left(1+z^{2}/2\right)$	0.537	0.0018
*λ*	−0.283	0.0037
Age	0.0231	0.00063
Age : $\log\left(1+z^{2}/2\right)$	0.0205	0.00039
Age : *λ*	−0.0604	0.00080

R^2^: 0.855; residual SD: 0.128 on 17,832 d.f.

Note: For an optimal design, the *SE_z_* curve should be flat, so *z* and *λ* need to be set to values that constrain the age coefficient to zero. The first three terms of the model then predict *SE_z_* for that design. Age is centred on 10 years.

Table 4 gives the optimal values for *λ* corresponding to the nine centiles in Figure 8. It confirms that optimal *λ* increases from 0.38 for the median to 0.90 for the 0.4th and 99.6th centiles, reflecting the different slopes of the median and outer centiles. It demonstrates that optimal sample composition depends on the centile used to calculate it. The median requires extra data in infancy, whereas the outer centiles need more uniformly sampled data. The function *optimal_design* in the appendix estimates optimal *λ* from *z* or vice versa.

**Table 4.** Optimal designs for sample composition, with n = 6878, where the values for *z* and *λ* substituted into Table 3 give a flat curve of *SE_z_* plotted against age.

Centile 100α	*z_α_*	*λ*	*SE_z_*
50th	0	0.38	0.041
25th_,_ 75th	±0.67	0.45	0.045
9th_,_ 91st	±1.33	0.60	0.054
2nd_,_ 98th	±2	0.76	0.066
0.4th_,_ 99.6th	±2.67	0.90	0.080

Note: The corresponding value of *SE_z_* is also given.

**Figure 9.**
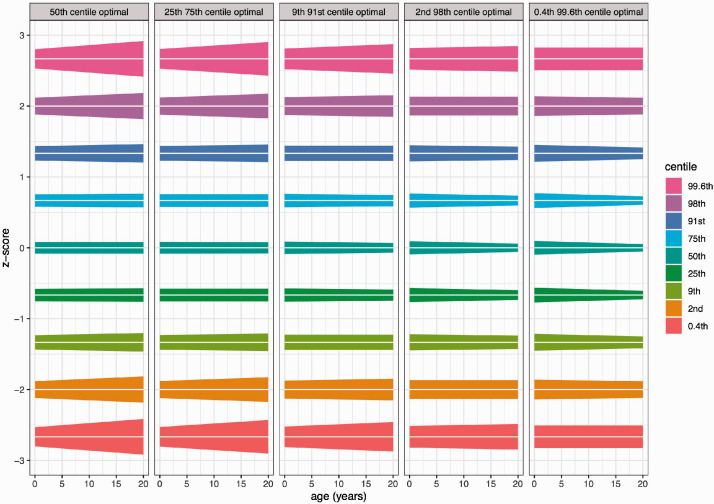
Stylised 95% confidence bands for the centiles, with n = 6878, showing the effect of varying the sample composition. The centiles are plotted on the z-score scale as horizontal straight lines, with wedge-shaped confidence bands and *SE_z_* curves assumed to be straight lines ignoring edge effects. For the centiles that define an optimal design, the wedge is a flat ribbon, i.e. the median in the left facet, the 25th and 75th centiles in the second facet, etc.

Figure 9 shows confidence bands for the nine centiles and how sample composition affects them. The centiles are plotted on the z-score scale, so each centile is a horizontal straight line with a wedge-shaped 95% confidence band. This is because the confidence interval for centile 100α is $z_{\alpha }\pm 2SE_{z}$ based on *n *=* *6878, and *SE_z_* is assumed linearly related to age, i.e. ignoring edge effects. So the confidence bands are wedge-shaped except when *λ* is optimal, in which case *SE_z_* is constant and the wedge is a ribbon. The left facet for example is optimal for the 50th centile and corresponds to *λ* = 0.38 (Table 4), so the 50th centile confidence band in that facet is a ribbon while the other bands are wedges. The other facets show confidence bands for other values of *λ* in Table 4 – in each case the optimal centile is a ribbon. Overall the figure highlights the quandary faced by the growth reference designer. It is not possible for all the bands to be flat ribbons, so which centile is best to use to define the sample composition? The Discussion addresses this question.

Point 6 above is puzzling: ‘the outer centile curves are progressively steeper in slope than the median curve’ – why should this be? The reason is that the median depends only on the *μ* curve, whereas the outer centiles depend also on the *σ* curve. The Results show that unlike the *μ* curve, the *σ* curve is relatively insensitive to age transformation, which means it is also largely unaffected by the sample composition. Centiles are of the general form $\mu +z_{\alpha }\sigma$ (equation (1)), so the dependence of *SE_z_* on *λ* is large for *μ* alone, requiring infant over-sampling; however, as $z_{\alpha }{\;}^{2}$ increases the influence of *σ* reduces the need for infant over-sampling, which in turn leads to a more uniform age distribution and larger optimal *λ*, as seen in Table 4.

**Figure 10.**
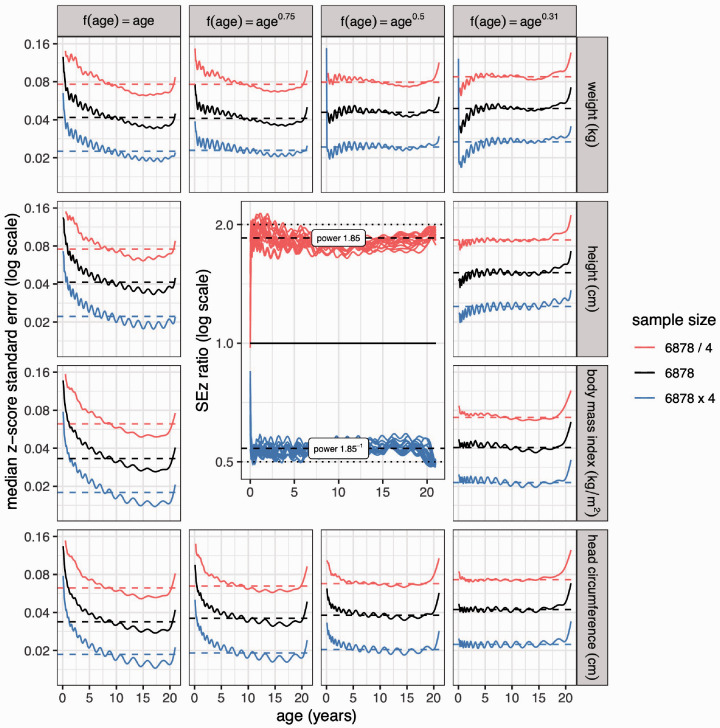
Z-score standard error curves for the median, by measurement and age power *λ* as in Figures 4, 5 and 7, showing the effect of a factor of four change in sample size, plotted on a log scale. In theory, $SE_{z}\propto \sqrt{1/n}$ (equation (3)) and $n\propto SE_{z}{\;}^{-2}$ (equation (5)), so the error should be doubled/halved. The inset shows the same curves as a ratio of each facet’s middle group curve, showing that $n\propto SE_{z}{\;}^{-1.85}$ rather than $n\propto SE_{z}{\;}^{-2}$.

Switching now from sample composition to sample size, *SE_z_* in Table 4 is based on a sample size of *n *=* *6878, and $SE_{z}{\;}^{2}$ varies inversely as *n* (equation (5)). So to achieve a given $SE_{z}$ tolerance, *n* needs to be scaled up or down appropriately. The validity of the power 2 for $SE_{z}{\;}^{2}$ is tested by simulating samples four times smaller and four times larger than 6878, and seeing how $SE_{z}$ varies; one would expect the factor of four to translate to, respectively, a doubling and halving of *SE*_z_. Figure 10, in the same format as Figures 7 and 8, shows the median $SE_{z}$ curves for GAMLSS models based on the three sample sizes, plotted on a log scale in facets by measurement and sample composition *λ*. Within each facet, the curves are roughly parallel and equally spaced, and they differ from the base curve by a factor close to 2. The inset to Figure 10 (which replaces the otherwise unremarkable central four facets) standardises the curves by dividing those in each facet by the middle curve values and superimposing the facets, showing that the multiplier is 1.85 (95% CI 1.83 to 1.87). So in detail, *n* varies as $SE_{z}{\;}^{-1.85}$ rather than $SE_{z}{\;}^{-2}$, or equivalently $SE_{z}$ varies as *n*^−1/1.85^ *= n*^−0.54^.

This allows the optimal sample size to be calculated based on the optimal sample composition. Two quantities are required: (a) the centile 100α on which to base the calculation, and (b) the required value of $SE_{z}$*_,_* call it $\hat{SE_{z}}$. Optimal *λ* is calculated from *z_α_* using Table 3, and then the resulting $SE_{z}$, e.g. in Table 4, which corresponds to *n *=* *6878, is scaled to match $\hat{SE_{z}}$ by setting the sample size to $n=6878\times {\left(SE_{z}/\hat{SE_{z}}\right)}^{1.85}$. The appendix function *optimal_design* does the calculation. The 95% confidence interval for *z_α_* is $z_{\alpha }\pm 2SE_{z}$, e.g. 0 ± 2 × 0.041 or (−0.082 to 0.082) for the 50th centile (see Table 4), and back-transformed this gives a 95% confidence interval for the 50th centile of (46.7 to 53.3). Table 5 gives confidence intervals for centiles from the 50th to the 0.4th_,_ based on optimally sampled datasets of size *n *=* *2000 to 93, 000.

**Table 5.** Optimal values of *SE_z_* and 95% confidence intervals for selected centiles, for different size samples from age 0 to 20 years.

Sample size	50th Centile (50) *λ* = 0.38			25th Centile (25.2) *λ* = 0.45			9th Centile (9.1)*λ* = 0.60			2nd Centile (2.3) *λ* = 0.76			0.4th Centile (0.38) *λ* = 0.90		
*N*	*SE_z_*	*C_low_*	*C_high_*	*SE_z_*	*C_low_*	*C_high_*	*SE_z_*	*C_low_*	*C_high_*	*SE_z_*	*C_low_*	*C_high_*	*SE_z_*	*C_low_*	*C_high_*
2000	0.08	44	56	0.087	20	31	0.11	6.1	13.1	0.13	1.2	4.1	0.16	0.15	0.93
3400	0.06	45	55	0.065	21	30	0.079	6.8	12.0	0.097	1.4	3.5	0.12	0.19	0.75
7200	0.04	47	53	0.044	22.5	28.1	0.053	7.5	11.0	0.065	1.7	3.1	0.078	0.24	0.60
12,000	0.03	47.6	52.4	0.033	23.2	27.4	0.040	7.9	10.5	0.049	1.8	2.9	0.059	0.27	0.54
26,000	0.02	48.4	51.6	0.022	23.9	26.7	0.026	8.3	10.0	0.032	1.9	2.6	0.039	0.30	0.48
93,000	0.01	49.2	50.8	0.011	24.6	26.0	0.013	8.7	9.6	0.016	2.1	2.5	0.020	0.34	0.43

Note: For each centile, the data are uniformly distributed on the optimal age*^λ^* scale. Confidence intervals for centiles above the 50th are obtained by difference from 100.

## 4 Discussion

‘How many children should I measure?’ is a common cry whenever growth reference centile projects are planned. This paper aims to provide an evidence-based framework to help researchers design such studies optimally.

The conventional way to calculate the sample size for reference centile studies is to specify the minimum precision required for one or more of the centile curves, and from this infer the number of individuals to measure per age group. The level of precision is typically specified as a standard error in measurement units, e.g. 0.3 cm for the 3rd height centile as used by Healy,^5^ or alternatively as a percentage or z-score. The first conclusion from the paper is that specifying the precision in z-score units leads to four major simplifications. First, the precision is essentially the same for all measurements, be they weight, height, BMI or head circumference. Second, the same precision applies to centiles that are equidistant above and below the median. Thus, one can require the 2nd centile to have a standard error of say 0.06 z-score units, and this will also apply by symmetry to the 98th centile.

The third advantage of the z-score scale is that the precision is broadly constant across age, and it can be made even more so by appropriately adjusting the age profile of the sample. This means that the subsidiary question: ‘How many children should I measure at each age?’ is as important as the sample size question. Optimising the age profile of measurements, i.e. the sample composition, makes the centile precision constant across the age range, which reduces the overall sample size needed to ensure the minimum required precision at any particular age. Choosing the sample composition comes down to deciding how much to over-sample in infancy compared to older ages, because infancy is the period of childhood that is least precise in centile terms. The paper proposes a simple though perhaps unintuitive way to define the sample composition, based on age raised to a suitable power, and the example below shows it in action.

The fourth advantage of working on the z-score scale is that the standard error and sample size are directly related – across the age range – such that *n* varies as SE_z_^−1.85^. One of the major uncertainties with centile construction is the impact on precision of the curve smoothing process. It is known that smoothing ‘borrows strength’ and hence increases the notional sample size,^31^ but by how much is unclear. So it is useful to compare the precision as described in the literature with what is achievable in practice. Healy^5^ calculated that to achieve *SE *=* *0.3 cm or *SE_z_* = 0.053 on the 97th height centile, 1000 children aged 8 were needed (1). Substituting the 97th centile (*z *=* *1.88) into the appendix function *optimal_design* gives optimal *λ* = 0.73 and *SE_z_* = 0.064 for *n *=* *6878, which scales up to *n *=* *9910 for *SE_z_* = 0.053. With *λ* = 0.73, 452 of the 9910 children are aged 7.5–8.5 (calculated using the *nagegp* function) compared to Healy’s 1000. So the required sample size is actually 452 not 1000, and the borrowed strength from the curve smoothing has increased the effective sample size by 2.3 times. The Cuban Growth Study^6^ with 28,000 children aged 0–20 was designed around Healy’s calculation, so on this basis it could have achieved the required precision with a sample size of around 13,000.

### 4.1 Steps to designing a growth reference centile study

This section explains the practical stages needed to design a cross-sectional growth reference centile study, using the *boys7482* data from birth to 20 years as example.

1. *Outcome measure*. First choose the primary outcome measure, the measurement to base the sample size calculation on. In practice, the choice is not critical for the reasons given above, but it is useful to be able to express the z-score precision in measurement units.
2. *Sample composition*. Next, decide which centile to use to define the required precision. Table 4 shows that the choice of centile critically affects the sample composition; the median for example needs an excess of infancy data (*λ* = 0.38), whereas the 0.4th or 99.6th centiles need ages close to uniformly distributed across the range (*λ* = 0.90). Figure 6 shows the corresponding age distributions. Clearly, the median requires extra infancy data, whereas the variability around the median that defines the outer centiles needs more data at older ages. Figure 9 is helpful here, showing the centile confidence bands for the different designs. The widest bands are for the 0.4th/99.6th and 2nd/98th centiles, but as argued earlier, few growth references use the outer centiles. In contrast, the bands for the median are much narrower. So to control the widest bands, a logical recommendation is to base the design on the 2nd/98th centiles, i.e. close to Healy’s 97th centile. This corresponds to *λ* = 0.76, i.e. moderate infant over-sampling.
3. *Precision*. Next, specify $\hat{SE_{z}}$ the required centile precision for the chosen centile 100α, building on the $SE_{z}$ values in Table 4. For the 2nd centile, the 95% confidence interval is given by $z_{\alpha }\pm 2SE_{z}$ back-transformed to (1.7 to 3.1). This is then scaled to the required $\hat{SE_{z}}$ by adjusting the sample size, as seen in Table 5. It is hard to recommend a particular sample size as there is a direct link between sample size and precision, which clearly depends on available financial resources. But that said, Table 5 shows that sample sizes between 7000 and 25,000 provide a reasonable compromise between economy and precision.
4. *Defining the age groups*. The *λ* value defines the sample composition in terms of how the measurement ages are distributed, and for survey design, the numbers need summarising in age groups. The age groups are assumed to be of equal width, e.g. whole years or fractions of a year, and the numbers for each group can be calculated using the *nagegp* function in the appendix. Take for example a study of 10,000 subjects in 20 one-year age groups from birth to 20 years, designed optimally based on the 2nd centile, where the numbers per year group fall from 1039 in infancy to 380 at age 19–20.
5. *Handling edge effects*. There is inevitably greater imprecision at the extremes of the age range (see e.g. Figure 8) and this is best handled by over-sampling the youngest and oldest age groups, perhaps by two or three times. A large sample post-puberty is important for linear measurements such as height where the frequency distribution plateaus in adulthood, and the adult centiles need to be parallel to each other. This in turn requires the CV and skewness curves to be flat in adulthood. Also, measurements can be collected at older ages, such as was done up to 23 years for the British 1990 reference,^32^ with the chart centiles truncated at a younger age.

### 4.2 Strengths and limitations

The study has several strengths. It is believed to be the first to systematically compare alternative sampling structures, in terms both of sample size and sample composition, and to quantify their impact on centile precision using simulations based on open source data. Against this there are several limitations, mainly to do with constraints on the analysis. The first is that the data are restricted to boys, so one cannot be certain that the conclusions apply to girls. However, there is no obvious reason why anthropometry for the two sexes should behave differently, so it reasonable to expect them to be similar. Second, the bootstrap analysis focuses on the GAMLSS BCCG family model (i.e. the LMS method) and not the kurtosis-extended BCT model. This is because the BCT model adds little to BCCG – its kurtosis adjustment affects only the most extreme centiles, beyond the 1st and 99th, and few growth charts have centiles extending that far.

A third limitation is that the analysis is restricted to data from birth to adult, and it does not consider studies over a shorter age range, for example 0 to 5 years. The optimal sample size cannot simply be scaled down based on the relative age ranges, e.g. *n* × 5/20, since the optimal sample composition from birth to adult is not uniform and the early life data are over-sampled. However, the appendix function *nagegp* can be used in this case, simply by specifying the required age range – see the example there. Remember though that the edge effects are relatively larger when the age range is smaller, and this needs taking into account as well.

Another design issue, which is not a limitation as such, is the role of longitudinal data in the context of constructing growth references. It is common to use such data from cohort studies for this purpose, which means treating the repeated data as cross-sectional. Such an approach does not invalidate the analysis *per se*, but it does need to be recognised that the precision is appreciably less than for truly cross-sectional data, being based on fewer subjects for the same sample size, and it may also be subject to bias. To advise on the calculation of sample size for longitudinal studies requires a separate research effort.

## 5 Conclusions

In conclusion, the study has addressed the longstanding problem of how to estimate the sample size for growth reference centile studies based on cross-sectional data. The main finding is that the analysis is best done on the measurement z-score scale, and that the required sample size can be defined in terms of the centile standard error expressed in z-score units. The sample composition needs to be optimised along with the sample size, to ensure that the required centile standard error is achieved across the age range, and a method for doing this is proposed.

## Dedication

The paper is dedicated to the memory of Professor Harvey Goldstein, a valued colleague whose many eminent statistical contributions included designing the 1972 Cuban Growth Study.

## Supplemental Material

sj-pdf-1-smm-10.1177_0962280220958438 - Supplemental material for Sample size and sample composition for constructing growth reference centiles Click here for additional data file. Supplemental material, sj-pdf-1-smm-10.1177_0962280220958438 for Sample size and sample composition for constructing growth reference centiles by TJ Cole in Statistical Methods in Medical Research
