# Supplementary material for: Sample size and sample composition for constructing growth reference centiles
Source: Stat Methods Med Res. 2020 Oct 12;30(2):488–507. doi: 10.1177/0962280220958438 (PMC8008444; doi:10.1177/0962280220958438)
Supplement: sj-pdf-1-smm-10.1177_0962280220958438 - Supplemental material for Sample size and sample composition for constructing growth reference centiles [file sj-pdf-1-smm-10.1177_0962280220958438.pdf]

Appendix: optimal design software

T J Cole

30 Sep 2020

R code for optimal design

This note describes two *R* functions for estimating optimal sample size and sample composition in growth reference centile studies.

First load the required libraries.

```
library(dplyr)
library(purrr)
library(ggplot2)
library(tibble)
library(sitar)
```

optimal_design

`optimal_design` calculates the optimal sample composition, based either on z or lambda, and the optimal sample size based either on N or SEz. If both N and SEz are given, N takes precedence. If both z and lambda are given, SEz also depends on age. The regression coefficients come from the model in table 3.

```
optimal_design <- function(z = -2, lambda = NA, N = NA, SEz = NA, age = 10) {
  stopifnot(!(is.na(z) && is.na(lambda)))
  N0 <- 6878
  coef <- c(`(Intercept)` = -3.0878266723299, age = 0.0231399511712584,
            fz2 = 0.536999348905583, lambda = -0.283052090744945,
            `age:fz2` = 0.0204776896422858, `age:lambda` = -0.0603665153280415)
  fz2 <- log(1 + z^2/2)
  if (all(is.na(lambda))) {
    lambda <- -(coef['age'] + coef['age:fz2'] * fz2) / coef['age:lambda']
  }
  if (all(is.na(z))) {
    fz2 <- -(coef['age'] + coef['age:lambda'] * lambda) / coef['age:fz2']
    z <- sqrt(2 * (ifelse(fz2 >= 0, exp(fz2) - 1, NA))) # inadmissible if lambda too small
  }
  SEz0 <- exp(coef['(Intercept)'] + coef['fz2'] * fz2 + coef['lambda'] * lambda +
    (age - 10) * (coef['age'] + coef['age:fz2'] * fz2 + coef['age:lambda'] * lambda))
  if (all(is.na(N))) {
    N <- N0
    if (all(is.na(SEz)))
      SEz <- SEz0
    else
      N <- N * (SEz0 / SEz)^1.85
  } else
    SEz <- SEz0 * (N0 / N)^(1/1.85)
  plo <- pnorm(z - 2 * SEz) * 100
  phi <- pnorm(z + 2 * SEz) * 100
```

```
  tibble(z = z, lambda = lambda, N = N, SEz = SEz, age = age, p = z2cent(z), plo = plo, phi = phi)
}
```

The arguments are as follows:

- z: the z-score on which to base the design. The default is -2 which equates to the 2nd centile, or if NA, optimal z is calculated from lambda.
- lambda: the power of age that defines the sample composition. The default NA means calculate optimal lambda from z.
- N: the total sample size per sex. The default NA means calculate from z or lambda, and SEz if provided.
- SEz: the target z-score standard error. The default NA means calculate from z or lambda, and N if provided.
- age: the age at which to calculate SEz. The default 10 returns mean SEz, and if z or lambda are optimal SEz is independent of age.

A tibble is returned with columns:

- z, lambda, N, SEz, age: defined as above.
- p: the centile corresponding to z.
- plo: lower 95% confidence interval for p.
- phi: upper 95% confidence interval for p.

Example

The following code estimates optimal lambda and SEz for each of the nine centiles in Figure 1 based on a sample of 10,000 children.

```
knitr::kable(optimal_design(z = -4:4*2/3, N = 10000), digits = c(2, 2, 0, 3, 0, 2, 2))
```

z	lambda	N	SEz	age	p	plo	phi
-2.67	0.90	10000	0.065	10	0.4th	0.26	0.56
-2.00	0.76	10000	0.054	10	2nd	1.75	2.93
-1.33	0.60	10000	0.044	10	9th	7.75	10.66
-0.67	0.45	10000	0.037	10	25th	22.97	27.64
0.00	0.38	10000	0.033	10	50th	47.34	52.66
0.67	0.45	10000	0.037	10	75th	72.36	77.03
1.33	0.60	10000	0.044	10	91st	89.34	92.25
2.00	0.76	10000	0.054	10	98th	97.07	98.25
2.67	0.90	10000	0.065	10	99.6th	99.44	99.74

nagegp

`nagegp` extends `optimal_design` by giving the numbers of measurements per age group, where the groups are of equal width.

```
nagegp <- function(z = -2, lambda = NA, N = NA, SEz = NA,
                   minage = 0, maxage = 20, n_groups = 20) {
  results <- optimal_design(z, lambda, N, SEz)
  if (is.na(lambda))
    lambda <- results$lambda
  if (is.na(N))
    N <- results$N
  N <- round(N / n_groups) * n_groups
  table <- tibble(n = N / n_groups,
    even = 1:(2 * n_groups + 1) %% 2 == 0, # TRUE design age, FALSE age group boundary
    age = seq(minage, # equal width groups
              maxage,
              length = length(even)),
    age0 = seq(minage^lambda, # unequal width groups
               maxage^lambda,
               length = length(even))^(1/lambda)) %>%
    mutate(n0 = age^lambda - lag(age^lambda, 2),
           n0 = ifelse(even, 0, n0),
           n0 = N * n0 / sum(n0, na.rm=TRUE),
           n0 = round(lead(n0))) %>%
    filter(even) %>% # age group means
    dplyr::select(n_varying = n0,
                  age,
                  n,
                  age_varying = age0)
  return(table)
}
```

The arguments are as follows:

- z, lambda, N, SEz: defined as above.
- minage: youngest age (default 0).
- maxage: oldest age (default 20).
- n_groups: number of age groups (default 20).

A tibble is returned with columns:

- n_varying, numbers for equal width age groups.
- age, mean ages for equal width age groups.
- n, number for each unequal width age group (only for longitudinal studies).
- age_varying, target ages for unequal width age groups (only for longitudinal studies).

Example 1

The following code calculates the age group sizes optimised for centiles from the 50th to the 99.6th (or equivalently from the 50th to the 0.4th), with a sample of 10,000 children from 0 to 20 years in one-year groups.

```
n_table <- map_dfc(0:4*2/3, ~{
  nagegp(z = .x, N = 10000) %>%
    select(!!z2cent(.x) := n_varying)
}) %>%
```

```r
  bind_cols(tibble(age = paste(0:19, 1:20, sep='-')), .)
knitr::kable(n_table)
```

age	50th	75th	91st	98th	99.6th
0-1	3172	2587	1662	1039	679
1-2	965	950	855	715	586
2-3	696	710	692	629	556
3-4	563	589	604	579	537
4-5	482	512	545	544	523
5-6	425	459	503	518	512
6-7	384	418	470	497	504
7-8	351	387	444	480	496
8-9	325	361	422	466	490
9-10	303	340	404	453	484
10-11	285	322	388	442	479
11-12	270	306	374	433	475
12-13	256	292	362	424	471
13-14	244	280	351	416	467
14-15	234	269	341	409	464
15-16	224	260	332	402	461
16-17	216	251	324	396	458
17-18	208	243	316	391	455
18-19	201	236	309	385	452
19-20	195	229	303	380	450

The table shows how the sample composition varies depending on which centile it is optimised for. Comparing the 50th and 99.6th centiles, the optimal number for the 50th centile is more than four times the size in the first year but less than half the size at age 19-20.

Example 2

`nagegp` can also be used to design studies over a narrower age range, e.g. 0-5 years. For example there are 3506 children in this age range in the table optimised for the 98th centile, and the following code returns the same group sizes (bar rounding):

```r
nagegp(z = 2, N = 3506, minage = 0, maxage = 5, n_groups = 5) %>%
  select(age, n_varying)
```

```
## # A tibble: 5 x 2
##     age n_varying
##   <dbl>     <dbl>
## 1   0.5      1038
## 2   1.5       715
## 3   2.5       629
## 4   3.5       579
## 5   4.5       544
```

This shows that the sample composition optimised for age 0-20 is also optimal for narrower age ranges.

Example 3

A third example contrasts the Cuban Growth Study design (as seen in Figure 4 of the paper) with the same design optimised to achieve the required precision. The study was powered to estimate the 3rd height centile of boys aged 8 with a standard error of 0.3 cm, which Healy (1974) showed required 1000 subjects. The final design increased the numbers by 10%.

Dividing the SE by the standard deviation of 5.75 cm gives SEz = 0.3 / 5.75 = 0.053. This is the required precision for boys aged 8, and for an optimal design the flat curve principle means it applies across the board. The code shows the age distribution for 0-20 years that was used and the corresponding design if it had been optimised.

```
tibble(age = c(1/6, 1/2, 5/6, 5/4, 7/4, 19/8, 25/8, 4:10,
                      c(22:29/2 - 1/4), 15:18, 77/4),
              n = c(rep(12, 3), rep(10, 4), 13, rep(11, 6), rep(6, 3),
                    rep(11, 5), 15, rep(8, 3), 13) * 100,
              span = c(rep(1/3, 3), rep(1/2, 2), rep(3/4, 2), rep(1, 7),
                       rep(1/2, 8), rep(1, 4), 3/2)) %>%
  ggplot(aes(x=age, y=n/span, width=span-0.02)) +
  xlab('age (years)') + ylab('frequency') +
  geom_bar(fill='gray', stat='identity') +
  geom_bar(aes(y=n_varying, width=NULL),
           data=nagegp(z = qnorm(0.03), SEz = 0.3 / 5.75) %>%
             mutate(n_varying = n_varying * 1.1),
           width=0.98, fill='gray50', stat='identity')
```

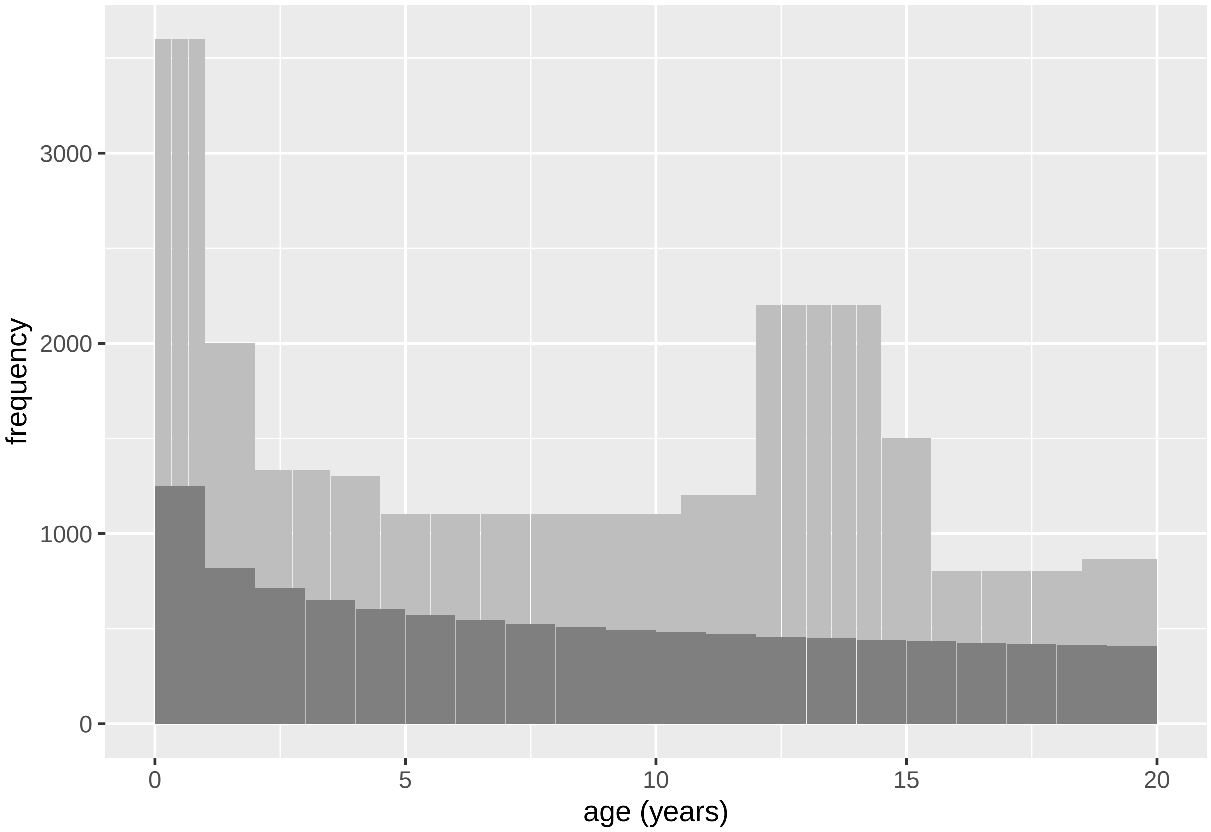

The figure highlights the saving in resources that optimisation can achieve. The sample size could have been reduced from 28,000 to around 11,000.
